# Artificial Intelligence and Machine Learning in Predicting Intradialytic Hypotension in Hemodialysis Patients: A Systematic Review

**DOI:** 10.7759/cureus.65334

**Published:** 2024-07-25

**Authors:** Taha Zahid Chaudhry, Mansi Yadav, Syed Faqeer Hussain Bokhari, Syeda Rubab Fatimah, Abdur Rehman, Muhammad Kamran, Aiman Asim, Mohamed Elhefyan, Osman Yousif

**Affiliations:** 1 Internal Medicine, Holy Family Hospital, Rawalpindi, PAK; 2 Internal Medicine, Pandit Bhagwat Dayal Sharma Post Graduate Institute of Medical Sciences, Rohtak, IND; 3 Surgery, King Edward Medical University, Lahore, PAK; 4 Internal Medicine, D. G. Khan Medical College, Dera Ghazi Khan, PAK; 5 Surgery, Mayo Hospital, Lahore, PAK; 6 Internal Medicine, Mayo Hospital, Lahore, PAK; 7 Medicine and Surgery, Jinnah Postgraduate Medical Centre, Karachi, PAK; 8 Internal Medicine, V. N. Karazin Kharkiv National University, Kharkiv, UKR

**Keywords:** ml, machine learning, ai, systematic review, artificial intelligence, renal, intradialytic hypotension, nephrology, hemodialysis, dialysis

## Abstract

Intradialytic hypotension (IDH) is a common and potentially life-threatening complication in hemodialysis patients. Traditional preventive measures have shown limited effectiveness in reducing IDH incidence. This systematic review evaluates the existing literature on the use of artificial intelligence (AI) and machine learning (ML) models for predicting IDH in hemodialysis patients. A comprehensive literature search identified five eligible studies employing diverse AI/ML algorithms, including artificial neural networks, decision trees, support vector machines, XGBoost, random forests, and LightGBM. These models utilized various features such as patient demographics, clinical data, laboratory findings, and dialysis-related parameters. The studies reported promising results, with several models achieving high prediction accuracies, sensitivities, specificities, and area under the receiver operating characteristic curve values for predicting IDH. However, limitations include variations in study populations, retrospective designs, and the need for prospective validation. Future research should focus on multicenter prospective studies, assessing clinical utility, and integrating interpretable AI/ML models into clinical decision support systems.

## Introduction and background

Intradialytic hypotension (IDH) is a common and potentially life-threatening complication affecting up to 30% of patients undergoing hemodialysis [1]. IDH is defined as a decrease in systolic blood pressure of ≥20 mmHg or a decrease in mean arterial pressure of ≥10 mmHg associated with clinical symptoms such as nausea, vomiting, abdominal cramps, muscle cramps, dizziness, or fainting [2]. The occurrence of IDH can lead to adverse consequences, including myocardial stunning, mesenteric ischemia, and an increased risk of vascular access thrombosis [3]. Moreover, IDH has been associated with an increased risk of hospitalization, cardiovascular events, and mortality in hemodialysis patients [4].

The etiology of IDH is multifactorial and involves factors such as excessive ultrafiltration, impaired cardiovascular reflexes, autonomic dysfunction, and endothelial dysregulation [5]. Traditional preventive measures, such as adjustments in dry weight, sodium profiling, and cooling dialysate, have shown limited effectiveness in reducing the incidence of IDH [6]. Therefore, there is a pressing need for more accurate and personalized approaches to predict and prevent IDH during hemodialysis sessions. In recent years, the fields of artificial intelligence (AI) and machine learning (ML) have gained significant attention in various medical applications, including the management of chronic kidney disease and hemodialysis [7]. Several studies have explored the use of AI/ML techniques to predict IDH in hemodialysis patients, utilizing diverse algorithms and feature sets derived from patient demographics, clinical data, and dialysis-related parameters [8-11].

This systematic review aims to comprehensively evaluate the existing literature on the use of AI/ML models for predicting IDH in hemodialysis patients. By synthesizing the findings from various studies, we hope to gain insights into the most effective AI/ML approaches, identify the key predictive features, and assess the potential clinical utility of these models in improving patient outcomes and reducing the burden of IDH.

## Review

Methodology

This systematic review has been reported according to the Preferred Reporting Items for Systematic Reviews and Meta-Analyses (PRISMA) guidelines, ensuring a rigorous and comprehensive evaluation of the included studies.

Search Strategy

A comprehensive literature search was performed using the following electronic databases: PubMed, Cochrane Central Register of Controlled Trials (CENTRAL), Hinari, and ScienceDirect. The search strategy was developed in consultation with a medical librarian and included a combination of relevant keywords and subject headings related to “artificial intelligence,” “machine learning,” “intradialytic hypotension,” “hemodialysis,” and “prediction models.” The searches were limited to studies published from the inception of the database to April 2024. Additional studies were identified through hand-searching the reference lists of relevant systematic reviews and included studies.

Eligibility Criteria

Studies were considered eligible for inclusion if they met the following criteria: (1) Original research studies with observational (cohort, case-control) or experimental (randomized controlled trials, quasi-experimental) study designs. (2) The study population comprised patients undergoing hemodialysis. (3) Utilization of AI or ML models to predict IDH. Studies were excluded if they (1) focused solely on descriptive or exploratory analyses without prediction models; (2) assessed general ML methods without specific application to IDH; (3) were case reports, reviews, editorials, or commentaries; or (4) were not published in English.

Study Selection

Two independent reviewers screened the titles and abstracts of all retrieved records to identify potentially eligible studies. Full texts were then obtained for all seemingly relevant records and independently assessed by the two reviewers for inclusion based on the eligibility criteria. Disagreements were resolved through discussion and consensus with a third reviewer.

Data Extraction

A standardized form was used to extract data from each included study by one reviewer and checked for accuracy by a second reviewer. Extracted information included study characteristics (author, year, country, study design), participant details (age, sample size, hemodialysis parameters), AI/ML model details (type of model, features used, training and validation methods), outcome measures (prediction accuracy, sensitivity, specificity, area under the curve (AUC)-receiver operating characteristic curve (ROC)) and key findings/conclusions.

Data Synthesis

Data synthesis was conducted through a narrative synthesis approach, as the heterogeneity of AI/ML models and outcome measures precluded a meta-analysis. The results are summarized in tables and discussed in the context of their implications for clinical practice and future research.

Results

Study Selection Process

**Figure 1:**
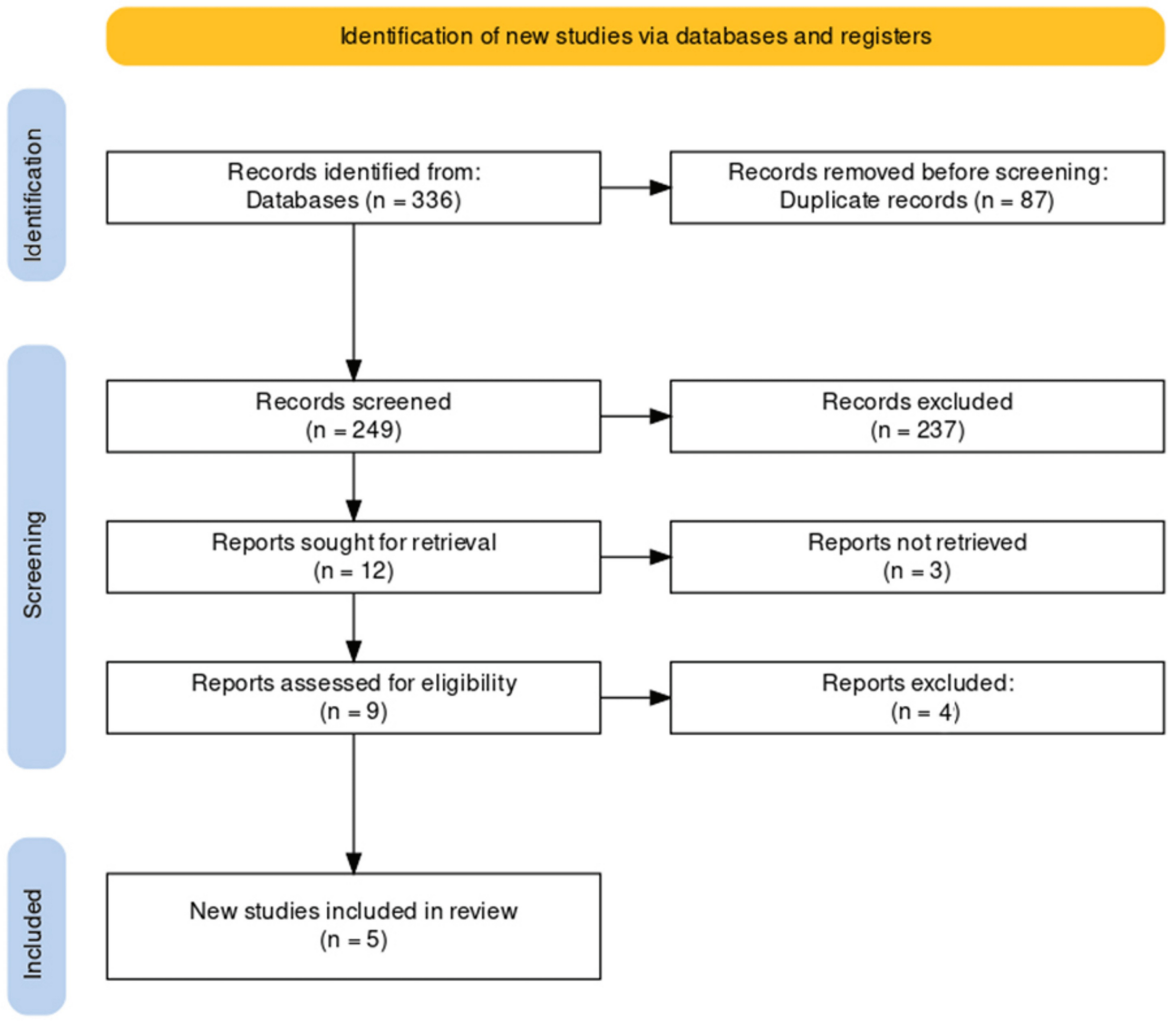
PRISMA diagram illustrating the study selection process. PRISMA: Preferred Reporting Items for Systematic Reviews and Meta-Analyses

The initial database search yielded 336 potentially relevant articles. After removing 87 duplicate records, the titles and abstracts of the remaining 249 publications were screened. Subsequently, after excluding 237 articles, the full text of the remaining 12 articles was assessed for eligibility, and five studies met the inclusion criteria. No additional eligible studies were identified through the reference list screening of the included articles. The entire study selection process is illustrated in the PRISMA flowchart (Figure 1).

Study Characteristics

The included studies employed various AI and ML models to predict IDH in hemodialysis patients. These studies were conducted across multiple countries, including Spain (n = 2), the United States (n = 1), and China (n = 2), between 2019 and 2023. All studies involved retrospective analyses of large datasets and dialysis records. The studies utilized diverse ML algorithms, such as artificial neural networks (ANNs), decision trees (DTs), support vector machines (SVMs), XGBoost, random forests (RFs), and LightGBM. The feature sets used for model development varied but commonly included patient demographics (age, gender, body weight), clinical data (primary disease, comorbidities, complications), laboratory findings (hemoglobin, albumin, electrolytes), and dialysis-related parameters (blood flow rate, arterial pressure, venous pressure, ultrafiltration rate) (Table 1).

**Table 1:** Characteristics of included studies.

Title	Author	Year	Country	Study type	Sample size
Development of an artificial intelligence model to guide the management of blood pressure, fluid volume, and dialysis dose in end-stage kidney disease patients: proof of concept and first clinical assessment	Barbieri et al. [11]	2019	Spain	Retrospective analysis	766,000 dialysis session
Predicting the appearance of hypotension during hemodialysis sessions using machine learning classifiers	Gómez-Pulido et al. [8]	2021	Spain	Retrospective analysis	98,015 hemodialysis sessions from 758 patients
Real-time prediction of intradialytic hypotension using machine learning and cloud computing infrastructure	Zhang et al. [9]	2023	USA	Retrospective analysis	693 patients, 42,656 hemodialysis sessions, 355,693 intradialytic SBP measurements
Construction of an early alert system for intradialytic hypotension before initiating hemodialysis based on machine learning	Hong et al. [12]	2023	China	Retrospective analysis	3,906 patients, 314,534 hemodialysis sessions
Machine learning-based intradialytic hypotension prediction of patients undergoing hemodialysis: a multicenter retrospective study	Dong et al. [10]	2023	China	Retrospective analysis	62,227 dialysis sessions

Barbieri et al. employed a multilayer ANN model with 60 features, achieving acceptable prediction accuracy for postdialysis weight, dialysis dose (Kt/V), minimum session systolic blood pressure, and postdialysis heart rate [11]. Gómez-Pulido et al. used DT and SVM classifiers with 22 clinical parameters, reporting accuracies of 74-81% and 74-80%, respectively, for predicting hypotension during hemodialysis sessions [8]. Zhang et al. developed a real-time XGBoost model for IDH prediction, utilizing 99 features and cloud computing infrastructure [9]. Their model achieved an area under the receiver operating characteristic curve (AUROC) of 0.89 for predicting hypotension 15-75 minutes in advance. Hong et al. constructed an early alert system using 18 ML algorithms, with the RF model demonstrating the best performance (AUROC = 0.812) for predicting IDH before initiating hemodialysis [12]. Dong et al. compared multiple ML models, including LightGBM, linear discriminant analysis (LDA), multilayer perceptron (MLP), XGBoost, and SVM, for predicting IDH [10]. They developed two models, IDH-A (27 features) and IDH-B (10 features), with LightGBM emerging as the superior model (AUROCs of 0.82 and 0.68, respectively).

The studies reported various performance metrics, such as prediction accuracy, precision, recall, F1-score, AUROC, sensitivity, and specificity, to evaluate the effectiveness of their ML models in predicting IDH (Table 2). However, it is important to note that the studies were conducted in different geographic regions, with variations in patient characteristics, dialysis protocols, and data collection practices, which could influence the generalizability of the findings.

**Table 2:** Summary of the studies included in the systematic review. ANN: artificial neural network; ME: mean error; MAE: mean absolute error; SBP: systolic blood pressure; HR: heart rate; IDH: intradialytic hypotension; DT: decision tree; SVM: support vector machine; BP: blood pressure; ML: machine learning; XGBoost: extreme gradient boosting; AUROC: area under the receiver operating characteristic curve; RF: random forest; LR: logistic regression; Hb: hemoglobin; CaPO_4_: calcium phosphate product; Na: sodium; K: potassium; PTH: parathyroid hormone; FER: ferritin; RCT: randomized controlled trial; LDA: linear discriminant analysis; MLP: multilayer perceptron; LightGBM: light gradient boosting machine; MAP: mean arterial pressure; UFR: ultrafiltration rate; UFV: ultrafiltration volume; RRT: renal replacement therapy

Author	Study focus	Hemodialysis parameters	AI/ML model	Data split	Model performance	Key predictors	Main findings	Conclusions
Barbieri et al. [11]	Predicting multiple hemodialysis endpoints	60 variables: patient characteristics, historical reactions, predialysis data, dialysis prescriptions	Multilayer ANN	70% training, 10% validation, 20% testing	Postdialysis weight: ME = 0.00 kg, MAE = 0.23 kg; Kt/V: ME = 0.00, MAE = 0.13; Min SBP: ME = -0.16 mmHg, MAE = 9.3 mmHg; Postdialysis HR: ME = 0.04 bpm, MAE = 7.3 bpm	Not specified	Acceptable prediction accuracy for multiple endpoints	The model can anticipate hemodynamic reactions and guide prescription adjustments
Gómez-Pulido et al. [8]	Predicting hypotension during hemodialysis	22 variables: demographics, interdialytic weight gain, urea clearance, blood flow, pressures, BP, HR	DT and SVM	70–95% training, 5–30% testing, 31 runs each	DT: accuracy = 75–81%, precision = >50%, specificity = >90%, low sensitivity; SVM: accuracy = 74–80%	Not specified	DT slightly outperformed SVM, up to 81% accuracy	Models could help prevent cardiovascular events during hemodialysis
Zhang et al. [9]	Real-time IDH prediction	99 variables: demographics, clinical data, treatment parameters, lab data, intradialytic vitals	XGBoost	80% training, 20% testing; session and patient randomization	AUROC = 0.89 (CI = 0.881–0.892); At threshold ≥0.09: sensitivity = 0.65, specificity = 0.90	Recent BP, prior hypotension rate, mean nadir BPs	Real-time IDH prediction feasible with good performance	Prospective studies needed to evaluate preventive interventions
Hong et al. [12]	Early IDH prediction	Demographics, clinical data, lab findings (Hb, albumin, CaPO_4_, Na, K, PTH, FER)	18 ML algorithms including LR, XGBoost, RF	80% training, 20% testing; 10-fold cross-validation	Accuracy = 74.0%, precision = 73.2%, recall = 66.9%, F1 score = 69.9%, AUROC = 81.2%	Pre-dialysis SBP, DBP, HR, dry weight, ultrafiltration capacity	19 parameters identified as predictive features; RF best performer	The early alert system could predict IDH before dialysis initiation
Dong et al. [10]	IDH prediction using two models	Gender, age, treatment time, weights, BP measures, MAP, UFR, UFV, RRT type, IDH history	LightGBM, LDA, MLP, XGB, SVM	70% training, 20% testing, 10% validation	IDH-A model: LightGBM (AUROC = 0.82) to SVM (AUROC = 0.61); IDH-B model: LightGBM (AUROC = 0.68) to SVM (AUROC = 0.49)	IDH history, HD-interval SBP mean, MAP mean, HD-interval SBP std	LightGBM is superior and interpretable	IDH-A and IDH-B models offer complementary risk prediction

Discussion

The present systematic review comprehensively evaluates the existing literature on the use of AI/ML models for predicting IDH in hemodialysis patients. The findings from the included studies highlight the potential of these modern computational approaches to address the longstanding challenge of IDH, which has been associated with adverse clinical outcomes and increased healthcare utilization [1,4]. Across the reviewed studies, a diverse range of AI/ML algorithms were employed, including ANNs, DTs, SVMs, XGBoost, RFs, and LightGBM [8-11]. This diversity underscores the versatility and adaptability of AI/ML techniques in tackling complex predictive tasks, as each algorithm operates on different principles and may be better suited for specific data structures or problem domains. The predictive models developed in these studies utilized a wide array of features, encompassing patient demographics, clinical data, laboratory findings, and dialysis-related parameters. This multimodal approach aligns with the multifactorial etiology of IDH, which involves factors such as excessive ultrafiltration, impaired cardiovascular reflexes, autonomic dysfunction, and endothelial dysregulation [3]. By incorporating diverse feature sets, these models can capture the intricate interplay of various risk factors and potentially provide more accurate and personalized predictions.

Several studies reported promising results, with high prediction accuracies, sensitivities, specificities, and AUROC values for predicting IDH [8-11]. These findings suggest that AI/ML models can effectively learn and leverage the complex patterns and relationships within the data to identify patients at high risk for developing IDH during hemodialysis sessions. However, it is essential to acknowledge the limitations of the reviewed studies. Most of the studies employed retrospective designs, which may introduce biases and limit the generalizability of the findings. Prospective validation studies in diverse patient populations and clinical settings are crucial to assess the real-world performance and clinical utility of these predictive models. Furthermore, the interpretability and transparency of AI/ML models remain a concern, as many algorithms operate as “black boxes,” making it challenging to understand the underlying decision-making processes. Future research should focus on developing interpretable AI/ML models or integrating interpretability techniques, such as feature importance analysis or local explanations, to enhance clinician trust and facilitate informed decision-making [13].

Another critical consideration is the integration of these predictive models into clinical decision support systems (CDSS) [14]. By seamlessly incorporating AI/ML models into existing electronic health record systems and clinical workflows, healthcare providers can leverage these advanced analytical capabilities to guide personalized treatment strategies and preventive measures for IDH. However, the successful implementation of such CDSS requires careful consideration of user experience, data privacy, and regulatory compliance. Looking ahead, multicenter prospective studies are warranted to validate the performance of AI/ML models in diverse clinical settings and patient populations. Additionally, comparative studies evaluating the relative strengths and weaknesses of different AI/ML algorithms for IDH prediction could provide valuable insights for algorithm selection and model optimization. Moreover, future research should explore the potential of combining AI/ML models with other technological advancements, such as wearable sensors or continuous blood pressure monitoring devices, to enable real-time prediction and timely interventions during hemodialysis sessions [15].

## Conclusions

The findings of this systematic review highlight the potential of leveraging AI/ML techniques for predicting IDH in hemodialysis patients. The included studies demonstrated promising results, with several AI/ML models achieving high predictive performance. However, several limitations should be considered, including variations in study populations, retrospective designs, and the need for interpretable and transparent models. Future research should focus on prospective, multicenter studies to validate the performance of AI/ML models in diverse patient populations and dialysis settings. Additionally, the integration of these predictive models into CDSS and their impact on clinical outcomes, patient quality of life, and healthcare resource utilization should be evaluated through randomized controlled trials. Efforts should be made to develop interpretable AI/ML models or to employ techniques such as feature importance analysis and local explanations to enhance the transparency and trust in these models. Furthermore, ensuring data quality and developing robust data imputation and preprocessing techniques are crucial to overcoming challenges related to inaccurate or missing data. Overall, the systematic review highlights the growing interest and potential of AI/ML techniques in predicting IDH, a critical step toward personalized and proactive management of this complication in hemodialysis patients.
